# Physicochemical, microbial, and aroma characteristics of Chinese pickled red peppers (*Capsicum annuum*) with and without biofilm†

**DOI:** 10.1039/d0ra00490a

**Published:** 2020-02-12

**Authors:** Qixian Zhang, Feng Zhang, Chuanjie Gong, Xinyi Tan, Yao Ren, Kai Yao, Qisheng Zhang, Yuanlong Chi

**Affiliations:** College of Biomass Science and Engineering, Healthy Food Evaluation Research Center, Sichuan University Chengdu 610065 P. R. China chiyl@scu.edu.cn +86 28 85404298 +86 28 85404298; Sichuan Dongpo Chinese Paocai Industrial Technology Research Institute Meishan 620020 P. R. China

## Abstract

Biofilm formation in the production of fermented vegetable might impact its quality and safety. In this study, physicochemical and microbial properties, volatile and aroma-active compounds between PRPs without biofilm (NPRP) and with biofilm (FPRP) were investigated by gas chromatography-mass spectrometry, gas chromatography-olfactometry, aroma extract dilution analysis, and spiking tests. The pH and titratable acidity were 3.66 ± 0.00 and 0.47 ± 0.08 g/100 g lactic acid in NPRP and 3.48 ± 0.01 and 0.87 ± 0.10 g/100 g lactic acid in FPRP, respectively. The nitrite level of the two PRPs was 1.87–1.92 mg kg^−1^, which was below the limited value (20 mg kg^−1^) of fermented vegetables regulated by the GB2760-2017. FPRP had relatively higher microbial and yeast numbers than NPRP, three common pathogens, namely, *Salmonella* spp., *Staphylococcus aureus*, and *Shigella* spp. were not detected. A total of 70 and 151 aroma compounds were detected in NPRP and FPRP, respectively, including 13 classes of compounds. The dominant aroma attributes of FPRP were sour, floral, mushroom-like, green, and smoky, while NPRP exhibits a mushroom-like flavor. Acetic acid, ethanol, α-terpineol, (*E*)-2-nonenal, 2-heptanol, phenylethyl alcohol, and linalool were potent key aroma-active compounds in NPRP and FPRP. Results of spiking tests showed that the addition of each substance not only increased its own odour, but also had significant effects on other smells. FPRP displayed richer varieties and contents of aroma profile than NPRP. However, some compounds, such as 4-ethylguaiacol and 4-vinylguaiacol, which were only detected in FPRP, had negative roles on the aroma attributes.

## Abstract 2

The aroma profile of PRPs was evaluated by GC-MS, GC-O, AEDA, OVA and spiking test. Biofilm can improve the variety and contents of aroma.

## Graphical abstract

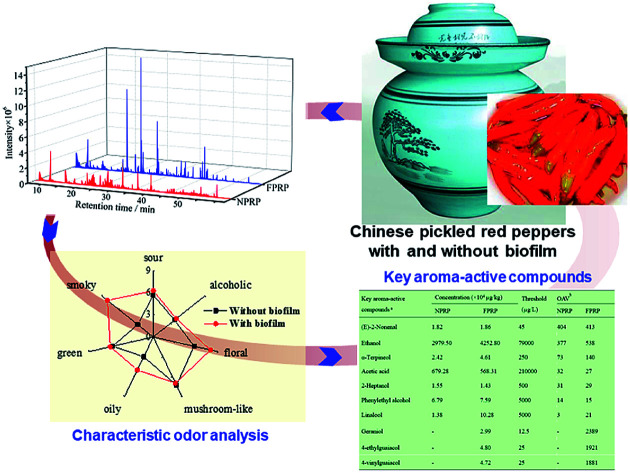

## 1. Introduction

Fermented foods, such as fermented sausage, yogurt, vinegar, wine, and fermented vegetables, produced or preserved by the action of microorganisms, are popular worldwide. During fermentation, a biofilm always appears on the surface of fermented foods and solutions. Biofilm formation depends not only on genetic bases and their regulation, but also on properties of the substratum and bacterial cells as well as environmental factors including pH, temperature and nutrient components.^1^ Biofilm consists of microorganisms and some chemical compounds, and the microorganisms in biofilm can secrete viscous metabolites that yield aggregated microbial cells. In general, flavor is an important index for the evaluation of fermented food. Biofilm helps convert substrates to acids and aids the generation of mellow products during vinegar brewing on account of protecting the acetic acid bacteria from damage caused by acetic acid.^2^ Similarly, it was proved that biofilm can increase the tolerance ability and metabolic activity of fermentation microorganisms.^3^ Formation of yeast biofilms was conducive to its resistance to external invasion, and promoted the fermentation of wild *Saccharomyces cerevisiae* strains of grape epidermis, which had an important impact on the color and flavor of wine.^4^ However, some fermented vegetables, like kimchi, was detected undesirable yeasty flavor coming from film-forming yeasts.^5^ What's more, film-forming microbes would change the chemical composition during the initial spoilage time in cucumber, thereby causing off-flavor and off-taste.^6^ These findings implied that biofilm exhibited differentiated roles on the flavor properties of varied fermented foods, and its effect mechanism on the flavor profile remains to be elucidated.

Fermented vegetables, as a common fermented food, are generally produced by immersing fresh vegetable in brine with certain salt concentration (4–15% w/w). Various fermented vegetables are found in different regions and countries, such as the fermented cucumbers in the USA,^7^ the fermented olives in the Mediterranean region,^8^ kimchi in Korea,^9^ and paocai in China.^10^ Biofilm formation on fermented vegetables and brine generally occurs.^11,12^ However, the effect of biofilm on the physicochemical, microbial, and aroma properties of fermented vegetables was infrequently reported. Thus, this topic needs intensive investigation.

Pickled red pepper (PRP), as one of the most typical fermented vegetables in southwest of China, was selected to represent fermented vegetables in the investigation of biofilm effect. In this study, the physicochemical and microbial properties and the aroma attributes of the PRP without biofilm (NPRP) and the PRP with biofilm (FPRP) were compared to illustrate the biofilm's influence. (1) pH and TTA values, NaCl and nitrite contents, bacteria and yeast counts and three common pathogens in food of NPRP and FPRP were measured. (2) Volatile compounds in NPRP and FPRP were detected using gas chromatography-mass spectrometry (GC-MS). (3) Aroma-active compounds in NPRP and FPRP were analyzed by gas chromatography-olfactometry/aroma extract dilution analysis. Furthermore, the potent key aroma-active compounds (PKAC) in NPRP and FPRP were quantified through combining internal standard with external standard methods and their odour activity value (OAV) was calculated to evaluate their contribution to flavor profile. (4) The actual contribution of PKAC was further verified through aroma spiking test.

## 2. Materials and methods

### 2.1. Preparation of PRP samples

All the materials for preparation of PRP were purchased from a local market in Chengdu, Sichuan China. Red peppers were washed and cut into small pieces (2 cm × 2 cm), and then 1000 g pepper was put into 2.5 L sterilized paocai jar. Brine with 10% NaCl salt concentrations was boiled. After cooled, it was added to the jars in a 1 : 1 proportion to the weight of red peppers.^13^ In the experimental group, 100 g of pickled red pepper was taken out every 1 month after opening. Then 100 g fresh red pepper of the same variety and boiled brine with 10% NaCl were added to the jar in a 1 : 1 proportion to the weight of red peppers according to the same method. In the control group, the jar was sealed with water to exclude air. Both jars were stored at an ambient temperature of 25 °C for ten months. The experimental group formed biofilm while the control group didn't. All PRP samples stored at 4 °C before use presented bright red color and typical fermented aroma.

### 2.2. Media and chemicals

Plate count agar, potato dextrose agar, xylose lysine desoxycholate agar, Baird–Parker agar, Luria–Bertani broth, and other reagents including NaCl, KH_2_PO_4_, glucose, and peptone were purchased from Land Bridge Biotechnology Co., Ltd. (Beijing, China). Phenylethyl alcohol, linalool, 2-heptanol, 4-ethylguaiacol, (*E*)-2-nonenal, α-terpineol, geraniol, ethanol, and acetic acid were purchased from Macklin (Shanghai, China). Heptanoic acid methyl ester was obtained from Sigma-Aldrich (Shanghai, China). The purity of all the above chemicals was higher than 98%. Other chemicals were of analytical grade and obtained from the Chron Chemical Reagent Co., Ltd. (Chengdu, China).

### 2.3. Physicochemical properties and microbial count determination

The pH was measured according to the method described in our previous study.^14^ PRP was cut into small pieces at approximately 1 cm × 1 cm, and a 20 g aliquot of minced PRP was mixed with 20 mL distilled water. The mixture pH was determined by a pH-3C meter (Yidian, Shanghai, China). The titratable acidity (TTA) was determined according to the titration method of AOAC 942.15 (Horwitz, 2000). Each test was performed thrice in parallel, and the results are expressed as the mean ± standard deviation.

The sodium chloride concentration was determined by titration with a 0.1 M silver nitrate solution, and potassium chromate was used as an indicator.^15^ The nitrite contents were determined according to the method described by Paik and Lee.^16^ NPRP and FPRP brines were evaluated for their nitrite contents with a L6S UV-vis spectrophotometer at 540 nm (Shanghai INESA Scientific Instrument Co., Ltd., Shanghai, China).

The numbers of microorganisms in the PRP samples were determined. A 25 g aliquot of minced PRP was homogenized in 225 mL of sterile saline containing 0.1% (w/v) peptone and 0.85% (w/v) NaCl by using a stomacher apparatus (Seward Medical, London, UK). Each sample was serially diluted in sterile saline and spread onto the plate count agar for total bacterial count, potato dextrose agar for yeast enumeration, xylose lysine desoxycholate agar for *Salmonella* spp. count, Baird–Parker agar for *Staphylococcus aureus* count, and Luria–Bertani broth for *Shigella* spp. enumeration. The potato dextrose agar plates were incubated at 28 °C for 3–5 d, and the others were incubated at 37 °C for 24–48 h.

### 2.4. Extraction of volatile compounds

Sample preparation and extraction of volatile compounds employed solid-phase microextraction (SPME) according to the methods described by Luo and Zhao with some minor modifications.^17,18^ A SPME sampler equipped with a 50/30 μm divinylbenzene/carboxen/polydimethylsiloxane fiber (DVB/CAR/PDMS) (Supelco Inc. Bellefonte, USA) was applied to extract the volatile compounds in PRP. Aliquots of 10.0 g of minced PRP were transferred into 20 mL gas-tight glass vessels (Agilent technologies, Santa Clara, USA). Prior to analysis, 20 μL of heptanoic acid methyl ester (0.05 mg mL^−1^ in methanol), as an internal standard, was added and mixed. After equilibration at 60 °C for 10 min, the sample was extracted with a DVB/CAR/PDMS fiber for 40 min at the same temperature. The fiber was then inserted into a GC injector port for 5 min to desorb the analytes. In all cases, the fibers were conditioned by inserting them into a GC injector port for 0.5 h at 270 °C before use and then subjecting them to desorption for 10 min at 270 °C between injections to prevent any contamination.

### 2.5. GC-MS analysis

The volatile compounds were analyzed using a GC-MS-QP2010 SE system (Shimadzu, Japan) equipped with an SH-Rxi-5Sil MS column (30 m × 0.25 mm × 0.25 μm, Shimadzu, Japan). The GC-MS conditions were achieved according to reported protocols with minor modifications.^18^ Helium was used as the carrier gas at a constant linear velocity of 1.0 mL min^−1^, and the sample extract was injected in a splitless mode. The GC temperature was programmed as follows: the column temperature was started at 40 °C for 3 min, increased to 130 °C at a rate of 5 °C min^−1^, and held for 5 min, then raised to 155 °C at 25 °C min^−1^, followed by increasing to 220 °C at 5 °C min^−1^, and held at 220 °C for 5 min. The injection temperature was 240 °C, and the ion source was at 220 °C. The mass spectrometer was operated in electron impact mode. The ionization energy, detector voltage, scan range, and scan rate were 70 eV, 350 eV, *m*/*z* 35–400, and 3 scans per s, respectively. The volatile compounds were identified based on comparing their retention indexes (RIs) and mass spectra with the standard database (NIST 14, Gaithersburg, MD, USA). The RIs were calculated using a C_7_–C_30_*n*-alkane series (Supelco Inc., Bellefonte, USA). The volatile compounds were semi-quantified through an internal standard method and heptanoic acid methyl ester (20 μL of 0.05 mg mL^−1^ in methanol) was used as an internal standard. The concentration of analyte (*C*, μg kg^−1^) was estimated as: *C* = (*k* × *m*_0_ × *S*)/*M*, where *m*_0_ and *M* are the weight of internal standard (μg) and sample (kg), respectively. *S* is the relative peak area (analyte/internal standard, %) and the response factor *k* was set as 1.

### 2.6. Gas chromatography-olfactometry (GC-O) and aroma extract dilution analysis (AEDA)

The GC system and conditions combined with a OP 275 sniffing port (Shimadzu, Japan) were the same as in Section 2.4. Olfactometry analysis was performed by three well-trained and experienced panelists (two females and one male, all with over 30 h of GC-O training) for odour detection and description.^19^ To maintain olfactory sensitivity and accuracy of panelists, the sniffing cone was purged with humidified air to reduce dehydration of the mucous membrane in the nasal cavity of the panelist.

The original aroma concentrate of PRP samples, extracted by SPME, was stepwise diluted by different split ratios, which varied from 1 : 2 to 1 : 64. The flavor dilution (FD) factor of a compound was defined as the reciprocal of the highest split ratio, in which it could be perceived with olfactometry. The AEDA for each sample was performed in triplicate, and the FD factor was obtained for each single odorant in NPRP and FPRP.

### 2.7. Quantification of PKAC and calculation of OAV

According to the results of GC-O and AEDA, the odorants with FD factors of 32 and higher than 32 were identified as PKAC. These compounds were quantified through the use of an internal standard (heptanoic acid methyl ester) and calibration curves of the corresponding aroma compound standards (as external standards). Standard solutions were prepared by dissolving authentic standard compounds in a model PRP solution (*i.e.*, containing 10% NaCl in distilled water). The calibration curves were obtained after triplicate analyses of each standard solutions at eight concentrations, which simulated the concentration ranges of various volatile compounds in PRP. When aroma compound standards were unavailable, the quantification was carried out using analogs, and the concentration of each compound was expressed as μg kg^−1^.

To evaluate the aroma potency of these PKAC, OAV was used and calculated as the concentration or the threshold in water, it's the ratio of the concentration to the threshold. Determination of the threshold value is described in Section 2.8.3.

### 2.8. Sensory evaluation, threshold value determination, and spiking test

#### 2.8.1 Sensory evaluation

Quantitative descriptive analysis was used to evaluate the differences of sensory aroma characteristics between the NPRP and FPRP samples. 15 candidates (aged 20 to 36, recruited from Food Engineering Department of Sichuan University) for the smell training once a week. Seven selected odours with different concentration gradients were prepared, and then with a capacity of 50 mL with cover PTFE holding 20 mL bottle with a good solution, and adopt different three digits marking (for example: the AAB, ABA, BAA, *etc.*). Three parallel samples were prepared for each selected flavor, and the same 2 cups were filled with 20 mL of tap water as a blank control. Requested to rate each concentration on a nine-point scale, with one for the weakest and nine for the strongest. According to the scoring situation, the personnel who were not sensitive to sniffing or whose scores were significantly different were eliminated, and 10 sensory evaluators (aged 20–27) were finally selected as members of the later sensory evaluation group. The training and experiment shall be in the sensory room at room temperature (23 ± 2 °C). The clean air exchanger shall make the room clean and free of odours.^20^ Selected candidates took part in sensory evaluation, threshold value determination, and spiking test. Seven attributes were generated to characterize the sensory properties of PRP: acetic acid (sour), ethanol (alcohol), geraniol (floral), 2-heptanol (mushroom-like), α-terpineol (oily), *E*-2-nonenal (green), and 4-ethylguaiacol (smoky).

#### 2.8.2. Determination of the threshold value of PKAC

Two alternative forced-choice sensory tests were used to determine the absolute threshold of the aroma's sensory perception, which was defined as the concentration corresponding to 80% correct response.^21^ Paired samples consisted of one test sample and a purified water sample. The test samples were prepared by serial dilutions of aroma compound standard in distilled water, thereby obtaining a series of concentrations of more than 3 μg L^−1^.

Two 20 mL samples, one containing purified water and one containing aroma compound, were randomly presented to each sensory panelist in plastic cups. The panelists smelt the sample and were asked to choose the one item they thought was more odorant than the others.

#### 2.8.3. Spiking test

To further verify the association of key odorants and the aroma perception of PRP, a spiking test based on sensory evaluation was performed. The FPRP and NPRP were spiked with a certain content of individual volatiles, and the concentration of added volatile compound was the same as its original concentration in PRP.^19^ The spiking sample and its original control were simultaneously subjected to sensory evaluation for the intensity of flavor index, as described in Section 2.8.1. The positive and negative effects indicated strong and weak intensities in the odour attributes, respectively. The assessors were asked to distinguish and assess the aroma attributes between the spiked sample and the unspiked one (control). All the tested compounds showed significant or extremely significant differences from the controls, thereby further indicating the important role of these key aroma-active compounds in the overall aroma of PRP. The effects of individual key odorants on the aroma perception in simulated food environment were distinguished, and the interactions among the sensory attributes were determined.

### 2.9. Statistical data analysis

One-way analysis of variance (one-way ANOVA) with a post-hoc Duncan's test was applied to compare the mean values by using SPSS20 (IBM Inc., New York, USA). In addition, the values were considered as significant difference and extremely significant difference when *p* < 0.05 and *p* < 0.01, respectively. Origin 9.0 (OriginLab Corporation, Northampton, USA) was applied to plot on the basis of data.

## 3. Result and discussion

### 3.1. Physicochemical and microbial properties and sensory evaluation of NPRP and FPRP

The indicators of pH and TTA are employed to evaluate the maturity of Chinese pickles, and the fermented vegetables were considered mature when pH was lower than 4 and TTA was higher than 0.3 g/100 g.^22^ As shown in Table 1, the pH and TTA were determined as 3.66 ± 0.00 and 0.47 ± 0.08 g/100 g for NPRP and 3.48 ± 0.01 and 0.87 ± 0.10 g/100 g for FPRP, respectively. This result indicated that these two PRPs have achieved maturity. FPRP had relatively higher microbial number than NPRP, the existence of biofilms enriched the microbial diversity in pickle system to some extent, which may increase the risk of nitrosamines and bioamines.^23^ The nitrite level of the two PRPs was 1.87–1.92 mg kg^−1^, which was below the limited value (20 mg kg^−1^) of fermented vegetables regulated by the Chinese National Food Standard regulation (GB2762-2017). Bacteria and yeast in NPRP and FPRP were in low levels of 10^3^ to 10^5^ colony-forming units per mL for microbial counts. In addition, three common pathogenic bacteria, namely, *Salmonella* spp., *S. aureus*, and *Shigella* spp., were not detected in NPRP and FPRP, which indicated that both NPRP and FPRP have high safety.

**Table 1:** Physicochemical properties and microbial counts of NPRP and FPRP^a^

Samples	pH	TTA (g/100 g lactic acid)	NaCl content (w/v, %)	Nitrite content (mg kg^−1^)	Microbial counts^b^ (log cfu mL^−1^)				
					Bacteria	Bacteria	*Salmonella* spp.	*S. aureus*	*Shigella* spp.
NPRP	3.66 ± 0.00^a^	0.47 ± 0.08^a^	9.93 ± 1.38^a^	1.87 ± 0.06^a^	2.93 ± 0.07^a^	2.47 ± 0.06^a^	nd	nd	nd
FPRP	3.48 ± 0.01^b^	0.87 ± 0.10^b^	10.01 ± 0.37^a^	1.92 ± 0.17^a^	4.09 ± 0.05^b^	3.97 ± 0.03^b^	nd	nd	nd

a Mean values in the same column with different letters indicate that they are significantly different at *p* < 0.05.

b nd represents no detection.

**Fig. 1:**
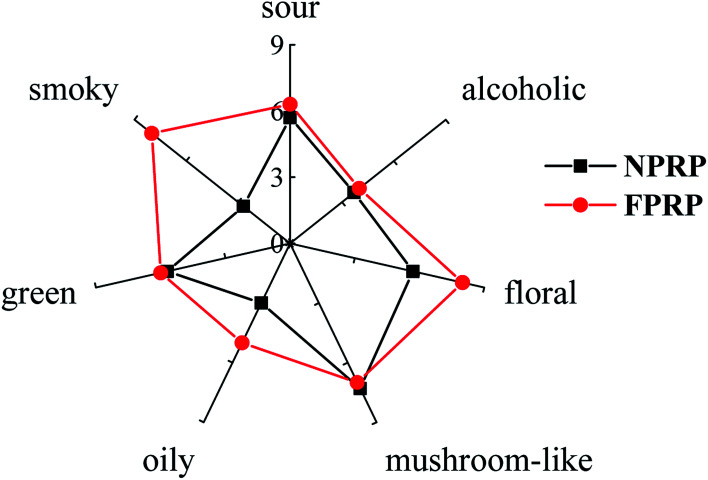
The characteristic odours of NPRP (■, black) and FPRP (●, red).

Seven characteristic odours were identified in NPRP and FPRP, namely, sour, alcoholic, floral, mushroom-like, oily, green, and smoky, as shown in Fig. 1. The dominant aroma attributes of FPRP were sour, floral, mushroom-like, green, and smoky, while they were mushroom-like, sour, floral, and green flavors for NPRP. FPRP exhibited richer flavor intensity compared with NPRP, especially for the floral, oily, and smoky odors (*p* < 0.05). However, a smoky odour, which is an unpleasant smell, was noted in FPRP instead of NPRP. This negative odour was also observed in wines and vegetables.^24^

### 3.2. Volatile profiles in NPRP and FPRP

**Fig. 2:**
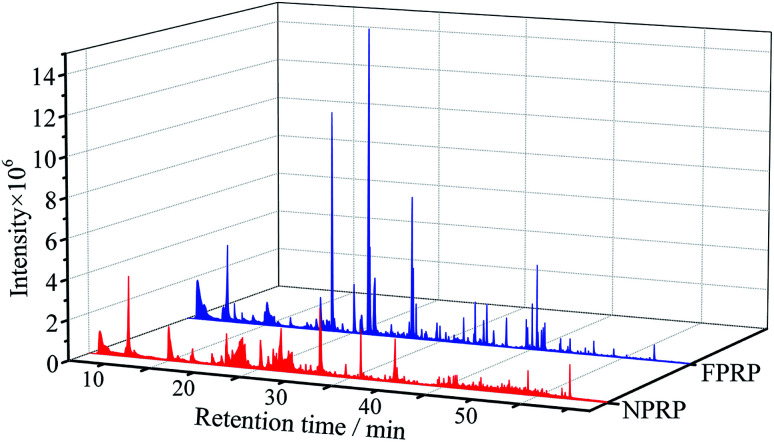
The total ion chromatography of volatile compounds in NPRP and FPRP.

**Fig. 3:**
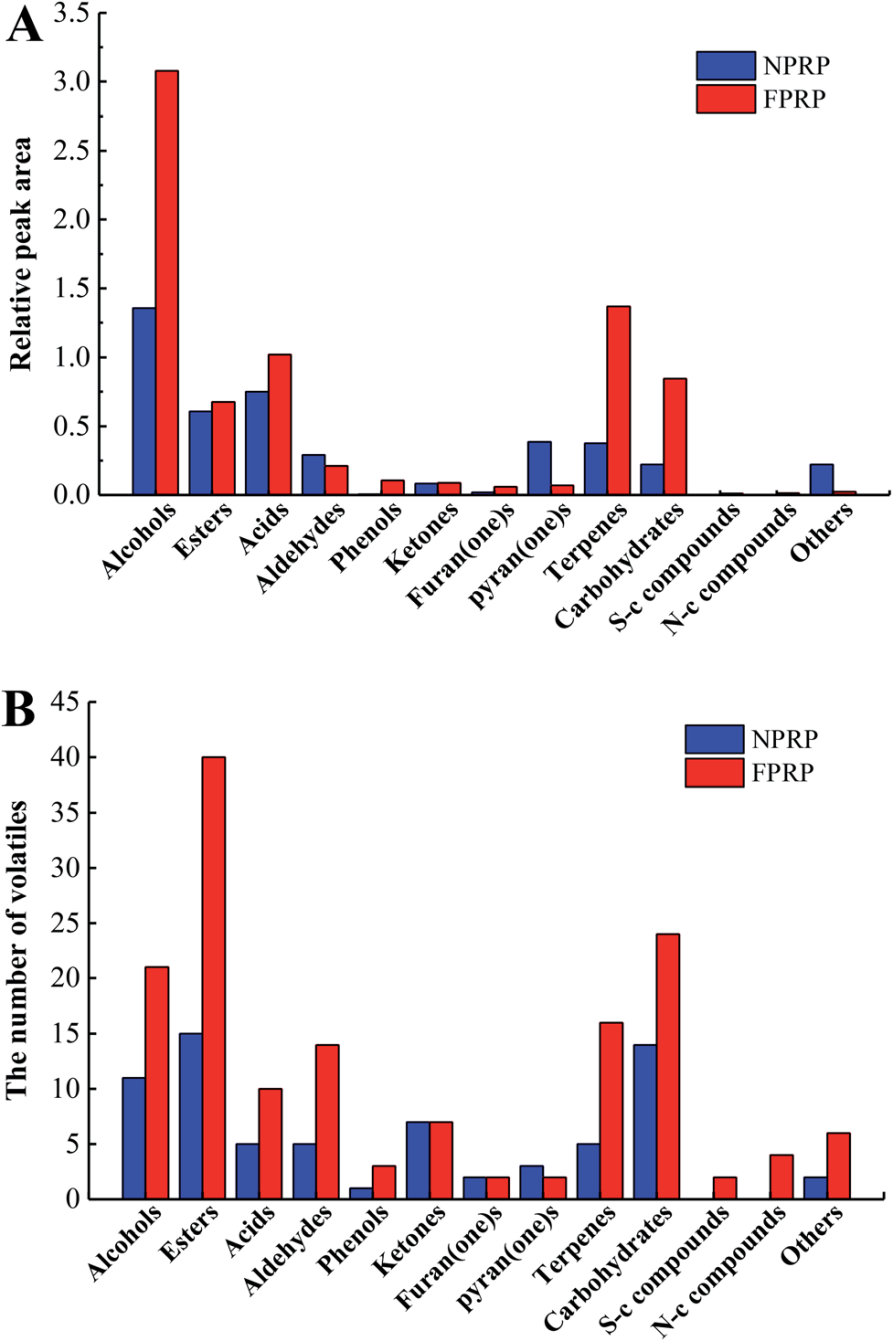
The relative peak area (A) and the number of volatiles (B) in NPRP and FPRP.

A total of 173 volatile compounds were detected and identified in NPRP and FPRP, as shown in Fig. 2 and ESI table.† 70 volatile compounds were found in NPRP, while 151 in FPRP. All volatiles were divided into 13 classes (Fig. 3). The 10 major classes of compounds were more abundant in FPRP than NPRP.

Alcohols had the highest content among the volatiles in these two PRPs. 21 types of alcohols were detected in FPRP, which was greater than those of NPRP (11 types). Ethanol, *n*-hexanol, and phenylethyl alcohol were the dominant alcohols in both PRPs. Alcohols, one of the main volatile compounds in fermented vegetables, came from the anabolic route of sugar *via* biosynthesis and the Ehrlich and Ribereau–Gayon metabolism of amino acids or the oxidation of lipids.^25^

Esters which contribute to fruit and floral aroma are generally considered important flavor components in fermented foods. Their types in the volatiles of NPRN and FPRP reached 15 and 40. Ethyl hexanoate, propyl 2,4-hexadiene carboxylate, and ethyl palmitate were the leading esters in NPRP, while ethyl hexanoate, 2-methylbutyrate, and ethyl decenoate were found in FPRP. 7 kinds of esters with the methyl groups were detected in FPRP, but no such esters were observed in NPRP. Esters with the methyl groups, which were derived from the catabolism of leucine and isoleucine, usually had low thresholds and would play an important role in flavor.^7^

In the acid group, acetic acid and (*E*)-3-hexenoic acid attained the highest content in NPRP, while acetic acid and hexanoic acid in FPRP. Acetic acid formed through the deaminase of amino acids or the hydrolysis of lipids in PRP.^26^ The formation of hexanoic acid was related to the β-oxidation of linoleic acid or the hydrolysis of ethyl hexanoate in red peppers. In addition, butyric acid, 2-methylbutyric acid, and 3-methylbutyric acid, which showed negative odors,^27^ were detected only in FPRP.

With regard to the minor volatile classes, aldehydes, phenols, furan(one)s, pyran(one)s, and sulfur-containing compounds also play important roles in the characteristic aroma of PRP. Nonanal was the dominant aldehyde volatile in NPRP and FPRP, benzaldehyde, octanal, and 4-(2,2-dimethyl-6-methylenecyclohexyl)butanal were only detected in FPRP at the same time. The major phenol 4-vinylguaiacol was found in the two PRPs, whereas 4-ethylguaiacol and 3,4-dimethylphenol were only observed in FPRP. These volatile phenols could come from the degradation of lignin or cell wall in peppers through microbial action.^28^ In addition, two sulfur-containing compounds, methional and 2-ethylthio ethylbenzene, were detected only in FPRP. These compounds cause an unpleasant flavor, which could be derived from the methionine degradation in the Maillard reaction.^29^

Huge difference of volatiles was observed between the FPRP and NPRP, only 48 volatiles were the same. The variety and content of flavor compounds were almost high in FPRP, suggesting a strong and rich odour. This outcome coincided with the results of the sensory evaluation, as shown in Fig. 1. The differences of flavor characteristics between the NPRP and FPRP could be correlated with the biofilm. The presence of biofilms enhanced the resistance of microorganisms in harsh environment and increased the number and type of microorganisms in the fermentation system.^11,30^ This result was confirmed through the microbial counts in NPRP and FPRP, as shown in Table 1. These organisms and their metabolism helped form the flavor compounds. Moreover, some extracellular polymeric substances, such as polysaccharide, protein, lipid, and nucleic acid, aggregated in the biofilm. These substances might affect the flavor in the sensorial way.^31^

### 3.3. Aroma-active compounds in NPRP and FPRP

According to the GC-O analysis, 32 aroma-active compounds, consisting of 11 esters, 6 alcohols, 4 terpenes, 4 acids, 2 pyrans(one)s, 2 furans(one)s, 2 phenols, and 1 aldehyde, were detected in NPRP and FPRP as shown in Table 2. Among these compounds, 8 aroma-active regions, namely, ethanol, α-terpineol, 2-heptanol, acetic acid, phenylethyl alcohol, (*E*)-2-nonenal (FD = 32), 2-ethenyl tetrahydro-2,6,6-trimethyl-2*H*-pyran (FD = 16), and 5-ethenyltetrahydro-5-trimethyl-2-furanmethanol (FD = 8), were both found with the same FD factor for NPRP and FPRP. Differences between the aroma profiles of these two samples were observed. Seven aroma-active regions, namely, ethyl hexanoate, nonanol, 9-decenoic acid, tetrahydro-2,2-dimethyl-5-(1-methyl-1-propenyl)-furan, propyl 2,4-hexadiene carboxylate, (*E*)-3-hexenoic acid and 3,7-dimethyl-oct-6-enoic acid, and ethyl ester, were obtained only in NPRP. These compounds contribute to the fruity, floral, fatty, citrus, and sweet odour. Otherwise, 4-ethylguaiacol (smoky, roast), 4-vinylguaiacol (smoky, burnt), geraniol (sweet, floral), 2-methylpentyl butyrate (sweet, apricot), hexyl caproate (apple-like), ethyl caproate (fruity, strawberry), and seven other compounds with an FD factor of ≤8 were detected only in FPRP. Notably, more odorants with high FD factor (≥16) were detected in FPRP than in NPRP. This outcome coincided with the results of flavor intensity revealed by sensory evaluation and GC-MS described above.

**Table 2:** Aroma-active compounds in NPRP and FPRP^a^

Aroma-active compounds	RI^c^ SH-Rxi-5Sil MS	Odour	Reference	FD factor		Methods of identification^d^
				NPRP	FPRP	
Linalool	1088	Floral, citrus-like	Zhu *et al.*, 2018 (ref. 32)	32	64	AB
Ethanol	<700	Alcoholic	Feng *et al.*, 2015 (ref. 33)	32	32	AB
α-Terpineol	1182	Oil, mint	Mehta *et al.*, 2017 (ref. 34)	32	32	AB
2-Heptanol	887	Mushroom-like, green	Mehta *et al.*, 2017 (ref. 34)	32	32	AB
Acetic acid	<700	Sour	Feng *et al.*, 2015 (ref. 33)	32	32	AB
Phenylethyl alcohol	1101	Floral, sweet	Feng *et al.*, 2015 (ref. 33)	32	32	AB
(*E*)-2-Nonenal	1146	Green, fruity, fatty	Pino & Quijano, 2012 (ref. 35)	32	32	AB
2-*E*thenyl tetrahydro-2,6,6-trimethyl-2*H*-pyran	955	Fresh, herbal	b	16	16	AB
Nonanol	1160	Floral, fatty	Liu *et al.*, 2018 (ref. 36)	16	nd	AB
5-Ethenyltetrahydro-5-trimethyl-2-furanmethanol	1074	Fresh, floral	Welke *et al.*, 2014 (ref.37)	8	8	AB
2-Phenylethyl acetate	1824	Floral, honey, fruity	Feng *et al.*, 2015 (ref. 33)	4	16	AB
Hexanol	852	Green, floral, fresh	Zhao *et al.*, 2017 (ref. 38)	2	8	AB
9-Decenoic acid	1323	Waxy, fatty, soapy	Welke *et al.*, 2014 (ref. 37)	2	nd	AB
3-Methyl-1-butanol	721	Malty, rancid	Feng *et al.*, 2015 (ref. 33)	1	4	AB
Tetrahydro-2,2-dimethyl-5-(1-methyl-1-propenyl)-furan	1032	Citrus, woody, minty	b	1	nd	AB
Propyl 2,4-hexadiene carboxylate	1085	Sweet, fruity	b	1	nd	AB
(*E*)-3-Hexenoic acid	1026	Fruity, cheesy	b	1	nd	AB
3,7-Dimethyl-oct-6-enoic acid, ethyl ester	1323	Fruity, citrus, sweet	b	1	nd	AB
4-Ethylguaiacol	1259	Smoky, roast	Feng *et al.*, 2014 (ref. 19)	nd	64	AB
4-Vinylguaiacol	1295	Smoky, burnt	Feng *et al.*, 2015 (ref. 33)	nd	32	AB
Geraniol	1248	Sweet, floral	b	nd	32	AB
2-Methylpentyl butyrate	1124	Sweet, apricot, banana-like	Giri *et al.*, 2010 (ref. 39)	nd	16	AB
Hexyl caproate	1300	Apple-like	b	nd	16	AB
Ethyl caproate	986	Fruity, strawberry	Zhu *et al.*, 2018 (ref. 32)	nd	16	AB
Hexyl acetate	1000	Fruity, floral	Welke *et al.*, 2014 (ref. 37)	nd	8	AB
3,6-Dihydro-4-methyl-2-(2-methyl-1-propenyl)-2*H*-pyran	1138	Oil, floral	Wüst *et al.*, 1999 (ref. 40)	nd	8	AB
9-Ethyl decenoate	1323	Floral	Welke *et al.*, 2014 (ref. 37)	nd	8	AB
(*Z*)-3,7-Dimethyl-1,3,6-octatriene (ocimene)	1034	Green, woody	Mehta *et al.*, 2014 (ref. 34)	nd	8	AB
Ethyl octanoate	1245	Fruity, floral, sweet	Duarte *et al.*, 2013 (ref. 41)	nd	4	AB
Hexanoic acid	1021	Rancidity, sheepskin	Zhao *et al.*, 2017 (ref. 38)	nd	1	AB
Ethyl phenylacetate	1226	Honey, floral, yeasty	Giri *et al.*, 2010 (ref. 39)	nd	1	AB

a nd represents no detection.

b Odour descriptions are based on the flavor database from www.thegoodscentscompany.com and https://pubchem.ncbi.nlm.nih.gov.

c Retention indices calculated on SH-Rxi-5Sil MS capillary column.

d Method of identification: by comparison of the mass spectrum (A) and RI (B) with the standard database (NIST 14, Gaithersburg, MD, USA).

Remarkably, some aroma-active compounds observed in this study had been previously reported in other fermented vegetables. Such as the most intense odorants in kimchi included dimethyl trisulfide, dially disulfide isomers, diallyl trisulfide, and methylallyl disulfide. In addition, 3-(methythio)propanal, (*E*,*Z*)-2,6-nonadienal, phenylacetaldehyde, linalool, (*E*,*E*)-2,4-decadienal, and 2,3-butanedione may play important roles in the formation of kimchi flavor.^42^*Trans*-4-hexenoic acid and *cis*-4-hexenoic acid were found in fermented cucumber.^43^ Ethyl acetate, 2-butanone, ethanol, isobutanol, and isopentanol dominated the volatile profile of fermented table olives.^44^ 7 main sulfur compounds (hydrogen sulfide, methanethiol, dimethyl sulfide, carbon disulfide, dimethyl disulfide, allyl isothiocyanate, and dimethyl trisulfide) and six organic compounds (methanol, ethanol, *n*-propanol, 2-propanol, acetaldehyde, and ethyl acetate) were detected in sauerkraut juice.^45^ However, the overall aroma profiles of picked red pepper were obviously different from those of fermented vegetables from Korea, the USA, Mediterranean region, and Europe. This result could be correlated with their raw material, process technology, local climate, and environment.

To evaluate each odorant in these two pickles, AEDA through a series of split ratios ranging from 1 : 2 to 1 : 64 was performed. Furthermore, the OAV, which determined the concentration and the odour threshold, was applied to verify the contributions of the potent key aroma-activity compounds (FD value ≥32) in NPRP and FPRP. As for NPRP, 19 aroma-active compounds were detected, and more than half of them had high FD factor (≥16). These odorants were composed of alcohols, aldehydes, acids, and esters. Seven compounds were identified as PKAC, namely, linalool, ethanol, α-terpineol, 2-heptanol, acetic acid, phenylethyl alcohol, and (*E*)-2-nonenal. According to the OAV evaluation in Table 3, (*E*)-2-nonenal, ethanol, α-terpineol, and acetic acid possessed the dominant contributions to the aroma profile of NPRP, exhibiting green, alcoholic, oil, and sour odour. As for FPRP, 25 aroma-active compounds were found, among which 10 odorants were PKAC. More complex aroma compounds, such as esters, phenols, and furans, were noted compared with NPRP. The formation of these compounds was related to the metabolism of microorganisms in biofilm.^46^ Three compounds with low concentrations, namely, geraniol, 4-ethylguaiacol, and 4-vinylguaiacol, contributed the most to the aroma profile due to their low thresholds. Geraniol had an OVA value of 2389 and contributed to sweet and floral aromas. The 4-ethylguaiacol and 4-vinylguaiacol, with the OAV values near 2000, could endow the smoky flavor even at low concentrations and have a negative impact on the FPRP flavor.^47^

**Table 3:** Concentrations, thresholds and odour activity value (OAV) of PKAC identified in NPRP and FPRP^a^

PKAC	RI1 SH-Rxi-5Sil MS	Concentration^b^ (×10^4^ μg kg^−1^)		Threshold (μg L^−1^)	OAV^c^	
		NPRP	FPRP		NPRP	FPRP
(*E*)-2-Nonenal	1146	1.82 ± 0.01^a^	1.86 ± 0.50^a^	45	404	413
Ethanol	<700	297.95 ± 36.12^a^	425.28 ± 14.41^b^	79 000	38	54
α-Terpineol	1182	2.42 ± 0.80^a^	4.61 ± 0.97^b^	250	73	140
Acetic acid	<700	679.28 ± 116.32^a^	568.31 ± 201.87^a^	210 000	32	27
2-Heptanol	887	1.55 ± 0.77^a^	1.43 ± 0.34^a^	500	31	29
Phenylethyl alcohol	1101	6.79 ± 2.69^a^	7.59 ± 1.10^a^	5000	14	15
Linalool	1088	1.38 ± 0.15^a^	10.28 ± 2.22^b^	5000	3	21
Geraniol	1248	nd	2.99 ± 0.94	12.5	nd	2389
4-Ethylguaiacol	1259	nd	4.80 ± 1.21	25	nd	1921
4-Vinylguaiacol	1295	nd	4.72 ± 1.07	25	nd	1881

a PKAC were the odorants with FD factors ≥32.

b Mean values in the same row with different letters indicate that they are significantly different at *p* < 0.05.

c OAV = concentration/threshold.^48^ nd represents no detection.

### 3.4. Effects of PKAC on the aroma attributes of NPRP and FPRP

A total of 7 and 9 PKAC were evaluated in NPRP and FPRP, respectively (Table 4). Spiking geraniol into FPRP provided an intense floral aroma, increased the mushroom-like and oily aromas, and significantly suppressed sour and smoky notes. Moreover, the mushroom-like attribute was easily influenced by the added odorants despite that those compounds, such as ethanol, linalool, α-terpineol, and geraniol, did not give off a mushroom-like odour. Adding α-terpineol could extremely significantly decrease the aromas of sour and mushroom-like in NPRP while causing an extremely significant reduction of the smoky note in FPRP. Therefore, the aromatic impact of each odorant was determined by its concentration and its interaction with its surrounding environment.

**Table 4:** Effects of PKAC on the aroma attributes of NPRP and FPRP

Samples	Positive	Negative
**NPRP**		
Ethanol	Alcohol^b^	—
Acetic acid	Sour^b^	—
2-Heptanol	Mushroom-like^a^, green^b^	Sour^a^
Linalool	Alcohol^a^, floral^b^	—
Phenylethyl alcohol	Floral^b^	—
α-Terpineol	Oily^a^	Sour^b^, mushroom-like^b^
(*E*)-2-Nonenal	Green^b^	Sour^b^, mushroom-like^b^

**FPRP**		
Ethanol	Alcohol^b^, mushroom-like^a^, oily^b^	Sour^a^, smoky^b^
Acetic acid	Sour^b^	—
2-Heptanol	Mushroom-like^b^, oily^b^, green^b^	Sour^b^, smoky^b^
Linalool	Mushroom-like^a^	Sour^a^, oily^b^, smoky^b^
Phenylethyl alcohol	Floral^b^, oily^b^	Smoky^b^
α-Terpineol	Mushroom-like^a^, oily^b^	Smoky^b^
(*E*)-2-Nonenal	Green^b^	—
Geraniol	Floral^a^, mushroom-like^b^, oily^b^	Sour^b^, smoky^b^
4-Ethylguaiacol	Smoky^a^	Sour^b^, floral^b^, mushroom-like^b^, oily^a^

a *p* < 0.05.

b *p* < 0.01.

## 4. Conclusion

The nitrite content of the PRP without biofilm (NPRP) and with biofilm (FPRP) was at a low level, and three common pathogens were not detected. The microbial population of FPRP was higher than that of NPRP, and the pH was lower, corresponding to the TTA. FPRP had stronger characteristic odors, richer variety, and higher contents of volatiles and aroma-active compounds compared with NPRP. However, some unpleasant odorants, such as 4-ethylguaiacol and 4-vinylguaiacol, were also present in FPRP. The PKAC in FPRP and NPRP exhibited visible synergism on the aroma. This study reveals the double-faced roles of biofilm on the flavor characteristics of Chinese PRP, which is a valuable discovery about biofilm function, and it could serve as a reference for the directional regulation and quality improvement of the flavor of fermented vegetables.

## Conflicts of interest

The authors state no conflict of interest.

## Supplementary Material

RA-010-D0RA00490A-s001
