# Supplementary material for: Physicochemical, microbial, and aroma characteristics of Chinese pickled red peppers (Capsicum annuum) with and without biofilm
Source: RSC Adv. 2020 Feb 12;10(11):6609–17. doi: 10.1039/d0ra00490a (PMC9049736; doi:10.1039/d0ra00490a)
Supplement: RA-010-D0RA00490A-s001 [file RA-010-D0RA00490A-s001.pdf]

This journal is © The Royal Society of Chemistry 2020

Supplementary Table Volatile compounds identified in NPRP and FPRP

Retention time/ min	Compounds	RI[1] SH-Rxi-5Sil MS	Concentration (μg/kg) (n=6) [2]		Methods of identification[3]
			NPRP	FPRP	
	Alcohols				
1	Ethanol	＜700	48.0±11.3	68.5±30.4	AB
2	3-Methyl-1-butanol	721	9.0±0.0	12.0±0.0	AB
3	(3-Methyl-2-oxiranyl)-methanol	740	-	77.0±0.0	AB
4	2,3-Butanediol	816	-	2.0±0.0	AB
5	1-Hexanol	852	38.0±17.0	95.0±120.2	AB
6	2-Heptanol	887	1.5±2.1	2.0±0.0	AB
7	6-Methyl-3-heptanol	949	-	1.0±0.0	AB
8	1-Octen-3-ol	968	0.5±0.7	-	AB
9	2-Ethyl-1-hexanol	1017	0.5±0.0	2.0±2.8	AB
10	2-Methyl-6-methylene-7-octen-2-ol	1058	-	18.0±12.7	AB
11	1-Octanol	1077	-	0.5±0.7	AB
12	4-Nonanol	1080	-	19.5±6.4	AB
13	3,7-Dimethyl-1,5,7-octatrien-3-ol	1092	-	8.0±11.3	AB
14	Phenylethyl alcohol	1101	35.0±4.2	42.0±4.2	AB
15	1-Nonanol	1160	0.4±0.0	0.5±0.7	AB
16	2,7-Dimethyl-2,7-octanediol	1165	-	0.5±0.7	AB
17	2,6,6-Trimethyl-bicyclo[3.1.1]heptan-3-ol,	1208	-	0.5±0.7	AB
18	(R)-3,7-Dimethyl-6-octen-1-ol	1213	-	1.5±2.1	AB
19	β,4-Dimethyl-3-cyclohexene-1-ethanol	1285	1.0±0.0	1.0±1.4	AB
20	2-Butyl-1-octanol	1457	-	0.5±0.7	AB
21	9-Dodecyn-1-ol	1489	1.0±1.4	0.1±0.2	AB
22	(E)-3,7,11-Trimethyl-1,6,10-dodecatrien-3-ol	1540	-	0.5±0.7	AB
23	6,11-Dimethyl-2,6,10-dodecatrien-1-ol	1551	0.5±0.7	-	AB
**Total**			**135.4**	**307.7**	
	Esters				
24	Propanoic acid, 2-hydroxy-, methyl ester	789	-	3.0±4.2	AB
25	Butyrolactone	908	-	0.2±0.2	AB
26	Hexanoic acid, 5-methyl-, methyl ester	924	-	1.0±1.4	AB
27	Pentanoic acid, 4-methyl-, ethyl ester	951	-	0.2±0.3	AB
28	Hexanoic acid, ethyl ester	986	-	13.0±4.2	AB
29	Hex-4-enoic acid, ethyl ester	987	19.5±4.9	-	AB
30	Acetic acid, hexyl ester	1000	5.5±0.7	4.0±1.4	AB
31	Propyl 2,4-hexadienecarboxylate	1085	6.0±0.0	-	AB
32	11-(2-Cyclopentenyl)undecanoic acid, ethyl ester	1096	-	0.1±0.1	AB
33	Octanoic acid, methyl ester	1122	-	0.1±0.2	AB
34	Butanoic acid, 2-methyl-, pentyl ester	1124	-	0.1±0.1	AB
35	Benzoic acid, ethyl ester	1156	-	0.5±0.7	AB
36	Methyl salicylate	1177	1.0±0.000	8.0±0.099	AB

37	Butanoic acid, 2-methyl-, hexyl ester	1185	-	8.0±11.3	AB
38	Butanoic acid, 3-methyl-, hexyl ester	1191	-	7.0±0.0	AB
39	2-Propenoic acid, 6-methylheptyl ester	1225	-	0.1±0.2	AB
40	2-Ethylhexyl acrylate	1225	-	0.1±0.0	AB
41	Benzeneacetic acid, ethyl ester	1226	-	1.2±1.1	AB
42	Acetic acid, 2-phenylethyl ester	1238	4.0±0.000	0.5±0.7	AB
43	Octanoic acid, ethyl ester	1245	-	1.7±1.8	AB
44	Propanoic acid, 2,2-dimethyl-, heptyl ester	1290	-	0.2±0.3	AB
45	Hexanoic acid, hexyl ester	1300	-	3.5±3.5	AB
46	3,7-Dimethyl-oct-6-enoic acid, ethyl ester	1323	3.0±1.4	0.2±0.3	AB
47	Ethyl 9-decenoate	1323	-	5.0±7.1	AB
48	Pentanoic acid, 2,2,4-trimethyl-3-hydroxy-, isobutyl ester	1340	-	0.5±0.7	AB
49	Propanoic acid, 2-methyl-, 2-ethyl-3-hydroxyhexyl ester	1353	0.2±0.2	0.5±0.7	AB
50	Decanoic acid, ethyl ester	1377	-	0.6±0.6	AB
51	Dodecanoic acid, methyl ester	1520	-	1.0±1.4	AB
52	2,2,4-Trimethyl-1,3-pentanediol diisobutyrate	1564	1.0±0.0	0.7±0.4	AB
53	Isopentyl 8-methylnon-6-enoate	1565	-	0.5±0.7	AB
54	Pentanoic acid, 2,2,4-trimethyl-3-carboxy isopropyl, isobutyl ester	1565	-	0.5±0.7	AB
55	Dodecanoic acid, ethyl ester	1569	2.0±0.0	1.5±0.7	AB
56	Pentadecanoic acid, 3-methylbutyl ester	1581	-	0.1±0.1	AB
57	Methyl tetradecanoate	1719	-	0.5±0.7	AB
58	Tetradecanoic acid, ethyl ester	1762	5.5±0.7	1.7±1.8	AB
59	Pentadecanoic acid, ethyl ester	1824	1.0±0.0	0.5±0.7	AB
60	1-Pentadecanol acetate	1838	0.5±0.7	0.1±0.1	AB
61	1,2-Benzenedicarboxylic acid, bis(2-methylpropyl) ester	1919	2.0±0.0	0.1±0.1	AB
62	Hexadecanoic acid, 15-methyl-, methyl ester	1920	-	0.5±0.7	AB
63	Ethyl 9-hexadecenoate	1934	1.0±0.0	0.4±0.2	AB
64	Hexadecanoic acid, ethyl ester	1955	8.5±2.1	3.5±0.7	AB
65	9,12-Octadecadienoic acid, methyl ester	2086	-	0.2±0.3	AB
**Total**			**60.7**	**67.6**	
	Acids				
66	Acetic acid	＜700	54.5±13.4	45.5±27.6	AB
67	Butanoic acid	790	-	0.5±0.7	AB
68	L-Lactic acid	813	-	0.5±0.7	AB
69	3-Methylbutanoic acid	870	-	0.5±0.7	AB
70	2-Methylbutanoic acid	878	-	1.5±2.1	AB
71	Hexanoic acid	1021	7.5±4.9	8.0±4.2	AB
72	(E)-3-Hexenoic acid	1026	5.5±7.7	-	AB
73	Heptanoic acid	1086	-	0.5±0.7	AB
74	Sorbic acid	1124	7.0±2.8	-	AB

75	Nonanoic acid	1264	-	0.1±0.2	AB
76	9-Decenoic acid	1323	0.5±0.7	0.3±0.3	AB
77	n-Hexadecanoic acid	1954	-	3.0±4.2	AB
**Total**			**75.0**	**102.0**	
	Aldehydes				
78	Hexanal	801	-	1.5±2.1	AB
79	(Z)-2-Heptenal	956	-	0.1±0.2	AB
80	Benzaldehyde	960	-	2.0±2.8	AB
81	Octanal	1004	-	2.0±2.8	AB
82	(E)-2-Octenal	1057	-	0.5±0.7	AB
83	phenylacetaldehyde	1071	-	1.5±2.1	AB
84	Nonanal	1091	25.5±34.6	5.0±7.1	AB
85	(E)-2-Nonenal	1146	0.7±0.5	1.0±0.0	AB
86	α,4-Dimethyl-3-cyclohexene-1-acetaldehyde	1163	2.0±0.0	1.0±0.0	AB
87	(Z)-(3,3-dimethylcyclohexylidene)-acetaldehyde,	1172	0.4±0.1	0.5±0.7	AB
88	Decanal	1191	0.5±0.7	0.5±0.7	AB
89	3-(2,6,6-Trimethyl-1-cyclohexen-1-yl)-2-propenal	1392	-	0.5±0.7	AB
90	4-(2,2-Dimethyl-6-methylenecyclohexyl)butanal	1479	-	4.5±0.7	AB
91	Tetradecanal	1650	-	0.5±0.7	AB
**Total**			**29.1**	**21.1**	
	Phenols				
92	3,4-Dimethylphenol	1164	-	4.0±5.7	AB
93	4-ethylguaiacol	1259	-	4.5±3.5	AB
94	4-vinylguaiacol	1295	1.0±0.0	2.0±2.8	AB
**Total**			**0.5**	**10.5**	
	Ketones				
95	5-Hepten-2-one	876	2.5±0.7	-	AB
96	3-Octanone	970	0.3±0.0	-	AB
97	4-Cyclopentylidene-2-butanone	1117	3.0±0.0	0.5±0.7	AB
98	5-Hydroxy-2,7-dimethyl-4-Octanone	1169		1.0±1.4	AB
99	1-(3-Methylphenyl)-ethanone	1170	0.1±0.1	-	AB
100	1-(4-Methylphenyl)-ethanone	1170	0.1±0.0	-	AB
101	1-(2,6,6-Trimethyl-1,3-cyclohexadien-1-yl)-2-buten-1-one	1360	1.5±0.7	3.5±2.1	AB
102	(Z)-1-(3,5,5-Trimethyl-2-cyclohexen-1-ylidene)-2-propanone	1379	1.0±0.1	-	AB
103	(Z)-6,10-Dimethyl-5,9-undecadien-2-one	1407	-	1.0±0.1	AB
104	2-Tridecanone	1493	-	1.5±2.1	AB
105	2-Pentadecanone	1693	-	1.0±0.1	AB
106	2-Hexadecanone	1694	-	1.0±0.1	AB
**Total**			**8.3**	**8.7**	
	Furan(one)s				
107	Dihydro-4-methyl-2(3H)-furanone	916	-	0.5±0.7	AB

108	Tetrahydro-2,2-dimethyl-5-(1-methyl-1-propenyl)-furan	1032	1.5±2.1	-	AB
109	cis-5-Ethenyltetrahydro-α,α,5-trimethyl-2-furanmethanol	1074	-	5.5±7.8	AB
110	3,6-Dimethyl-2,3,3a,4,5,7a-hexahydrobenzofuran	1173	0.2±0.3		AB
**Total**			**1.7**	**6.0**	
	pyran(one)s				
111	2-Ethenyltetrahydro-2,6,6-trimethyl-2H-pyran	955	7.0±0.4	3.5±0.7	AB
112	3,6-Dihydro-4-methyl-2-(2-methyl-1-propenyl)-2H-pyran	1138	14.0±0.0	3.5±0.7	AB
113	2-Hydroxy-3-acetyl-6-methyl-4-pyrone	1342	24.0±7.1	-	AB
**Total**			**38.7**	**7.00**	
	Terpenes				
114	D-Limonene	1016	-	35.0±0.0	AB
115	α-Pinene	1023	-	0.5±0.0	AB
116	trans-β-Ocimene	1023	-	0.5±0.0	AB
117	(Z)-3,7-Dimethyl-1,3,6-octatriene,	1034	-	2.0±1.4	AB
118	trans-Linalool oxide (furanoid)	1059	19.0±2.8		AB
119	Linalool	1088	2.0±0.0	89.5±112.4	AB
120	2,6-Dimethyl-5,7-octadien-2-ol	1152	-	=1.0±1.4	AB
121	α-Terpineol	1182	13.0±17.0	34.5±21.9	AB
122	β-copaene	1204	-	1.0±1.4	AB
123	2,7-Dimethyl-2,6-octadien-1-ol	1211	-	0.5±0.7	AB
124	Citronellol	1213	-	2.0±2.8	AB
125	Geraniol	1248	-	0.5±0.7	AB
126	Nerol	1249	-	0.5±0.7	AB
127	cis-Thujopsene	1432	-	0.2±0.3	AB
128	α-Santalol	1446	0.6±0.5	0.5±0.7	AB
129	α-Guaiene	1466	3.0±1.4		AB
130	β-Vatirenene	1470	-	7.0±9.9	AB
131	Nerolidol	1557	-	0.5±0.7	AB
**Total**			**37.6**	**136.7**	
	Carbohydrates				
132	1,3,5,7-Cyclooctatetraene	889	-	2.5±3.5	AB
133	2,6-Dimethyl-1,6-octadiene	976	2.5±3.5	1.5±2.1	AB
134	Hexylidene-cyclopropane	976	-	4.0±5.7	AB
135	4,6-Decadiene	991	1.5±2.1	-	AB
136	1-Methyl-4-(1-methylethenyl)-cyclohexene	1016	2.0±1.4	-	AB
137	1-Decen-3-yne	1036	-	0.5±0.7	AB
138	1-Methyl-4-(1-methylethylidene)-cyclohexene	1071	0.5±0.7	-	AB
139	3-Methyl-6-(1-methylethylidene)-cyclohexene	1072	-	2.5±3.5	AB
140	7-Ethenyl-5-undecene	1084	-	0.5±0.7	AB
141	1-Pentyl-cyclopentene	1084	-	0.3±0.4	AB

142	Naphthalene	1183	-	0.2±0.3	AB
143	2,7,7-Trimethyl-bicyclo[2.2.1]hept-2-ene	1229	-	0.1±1.4	AB
144	1,2,3,4-Tetrahydro-1,1,6-trimethyl-naphthalene	1292	-	0.1±0.2	AB
145	1-Methylnaphthalene,	1294	-	0.1±0.1	AB
146	Benzocycloheptatriene	1294	-	0.1±0.1	AB
147	Tridecane	1299	-	0.1±0.1	AB
148	Megastigma-4,6(Z),8(E)-triene	1320	-	5.5±7.8	AB
149	(1R,2S,7R,8R)-2,6,6,9-Tetramethyl-tricyclo[5.4.0.0(2,8)]undec-9-ene	1332	1.5±0.7	0.5±0.7	AB
150	2-Methyl-tridecane	1346	4.0±1.4	5.5±6.4	AB
151	Isosativene	1356	1.0±0.0	-	AB
152	Tetradecane	1385	1.0±0.0	5.5±6.4	AB
153	1,1-Dimethyl-2-nonyl-cyclopropane,	1435	1.0±1.4	-	AB
154	2-Methyl-1-tetradecene	1436		3.0±4.2	AB
155	2,4a,5,6,7,8,9,9a-Octahydro-3,5,5-trimethyl-9-methylene-1H-benzocycloheptene	1439	2.0±0.0	2.5±3.5	AB
156	2-Methyl-tetradecane	1452	1.0±1.4	3.5±5.0	AB
157	4,5-Di-epi-aristolochene	1469	1.0±1.4	24.0±33.9	AB
158	3-Tetradecyne	1479	2.0±0.0	2.0±2.8	AB
159	7-Tetradecyne	1489	1.0±1.4	-	AB
160	Guaia-1(10),11-diene	1492	-	19.0±26.9	AB
161	2-Methyl-3-undecene	1617	-	0.2±0.2	AB
**Total**			**22.0**	**84.5**	
	Sulfur-containing compounds				
162	Methional	905	-	0.5±0.7	AB
163	[2-(ethylthio)ethyl]-Benzene	1317	-	0.5±0.7	AB
**Total**			**0.0**	**1.0**	
	Nitrogen-containing compounds				
164	2-Hydroxy-3-cyanopyridine	1190	-	0.5±0.7	AB
165	1-Methyl-2-nitro-benzene	1216	-	0.5±0.7	AB
166	N-[4-Bromo-n-butyl]-2-piperidinone	1660	-	0.1±0.2	AB
167	3-Amino-5-(4-morpholinylmethyl)-2-oxazolidinone	1855	-	0.1±0.2	AB
**Total**			**0.0**	**1.2**	
	Others				
168	Ethyl maltol	1184	7.0±0.0	-	AB
169	5-Methoxyindane	1272	1.5±1.4	0.2±0.3	AB
170	3-Ethenyl-2,3-dihydro-1,1-dimethyl-1H-indene	1347	-	0.2±0.2	AB
171	1-Ethenyl-1-methyl-2,4-bis(1-methylethenyl)-cyclohexane	1386		0.5±0.7	AB
172	Decahydro-1,1,7-trimethyl-4-methylene-1H-cycloprop[e] azulene	1424	-	0.5±0.7	AB
173	1,2,3,3a,4,5,6,7-Octahydro-1,4-dimethyl-7-(1-methylethenyl)-azulene	1453		0.5±0.7	AB

**Total**	**22.0**	**2.3**

[1] Retention indices calculated on SH-Rxi-5Sil MS capillary column.

[2] Relative percentage to that of internal standard (0.05 mg/mL, 20 µL heptanoic acid methyl ester in methanol) using SH-Rxi-5Sil MS capillary column. Values expressed as average (n=6) ±standard deviation, - represents not detected.

[3] [d] Method of identification: by comparison of the mass spectrum (A) and RI (B) with the standard database (NIST 14, Gaithersburg, MD, USA).
